# Supplementary material for: Eggerthella lenta evades bacteriophage through reversible megabase-scale inversions of capsular polysaccharide gene clusters
Source: Res Sq. 2026 May 5:rs.3.rs-9488777. Preprint. [Version 1] doi: 10.21203/rs.3.rs-9488777/v1 (PMC13174815; doi:10.21203/rs.3.rs-9488777/v1)
Supplement: Supplement 1 [file NIHPPrs9488777v1-supplement-1.pdf]

SUPPLEMENTAL FIGURES AND LEGENDS

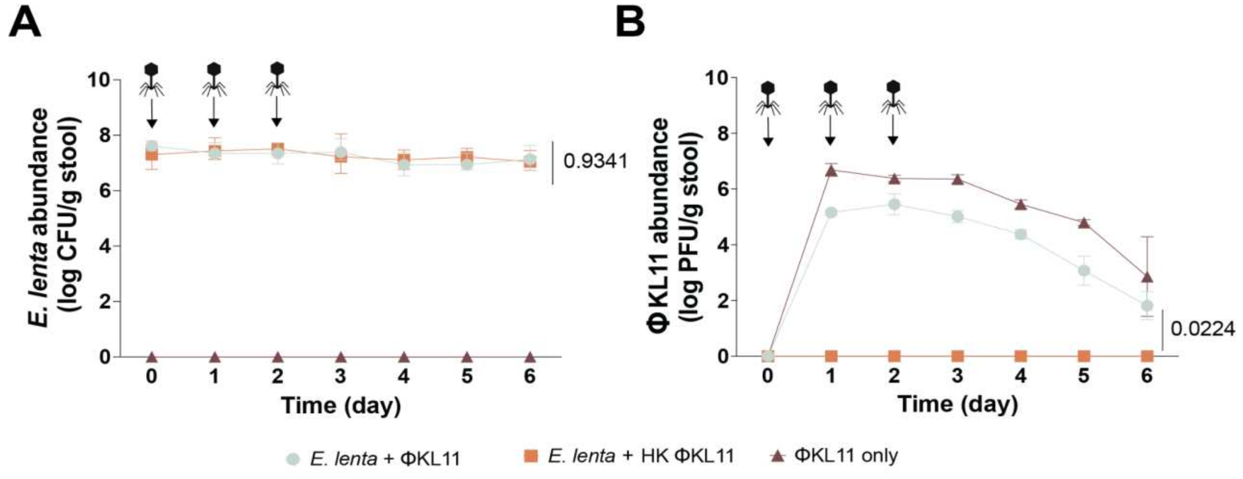

Figure S1. *E. lenta* evades phage predation in the mouse gut in a repeated experiment. (A–B) Germ-free (GF) BALB/c mice were monoassociated with *E. lenta* DSM2243 for one week prior to phage treatment. Mice then received either heat-killed (HK) or active ΦKL11 by oral gavage for three consecutive days. A GF control group that received active ΦKL11 only was also included (n = 3-4 mice per group). **(A)** Longitudinal stool measurements of *E. lenta* abundance (CFU/g) and **(B)** ΦKL11 abundance (PFU/g). *p*-values, two-way ANOVA.

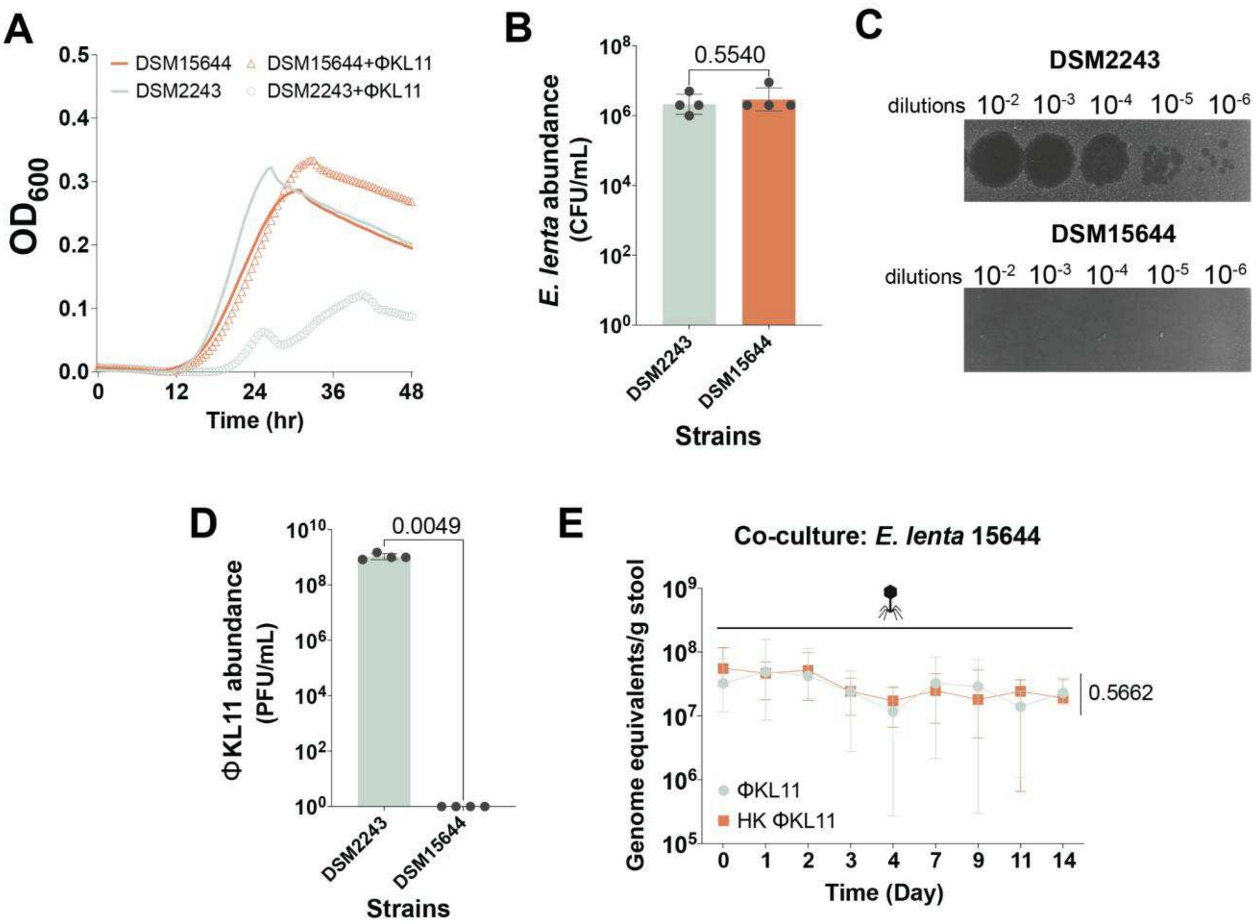

Figure S2. *E. lenta* evades phage predation in a co-culture experiment in GF mice (A) Growth curves of *E. lenta* DSM2243 and *E. lenta* DSM15644 in the presence or absence of ΦKL11. Curves represent the mean of *n*=4 biological replicates. **(B)** *E. lenta* cell counts for DSM2243 and DSM15644 cultures used in the plaque assay in **Fig. S2C**. **(C-D)** ΦKL11 robustly lyses *E. lenta* DSM2243 on an agarose overlay but does not lyse *E. lenta* DSM15644. **(E)** *E. lenta* DSM15644 abundance measured by qPCR during co-colonization with the ΦKL11-sensitive *E. lenta* DSM2243 (1:1 inoculation; n=9–10 mice per group). *p*-values, Welch's *t* test **(B,D)** and two-way ANOVA **(E)**.

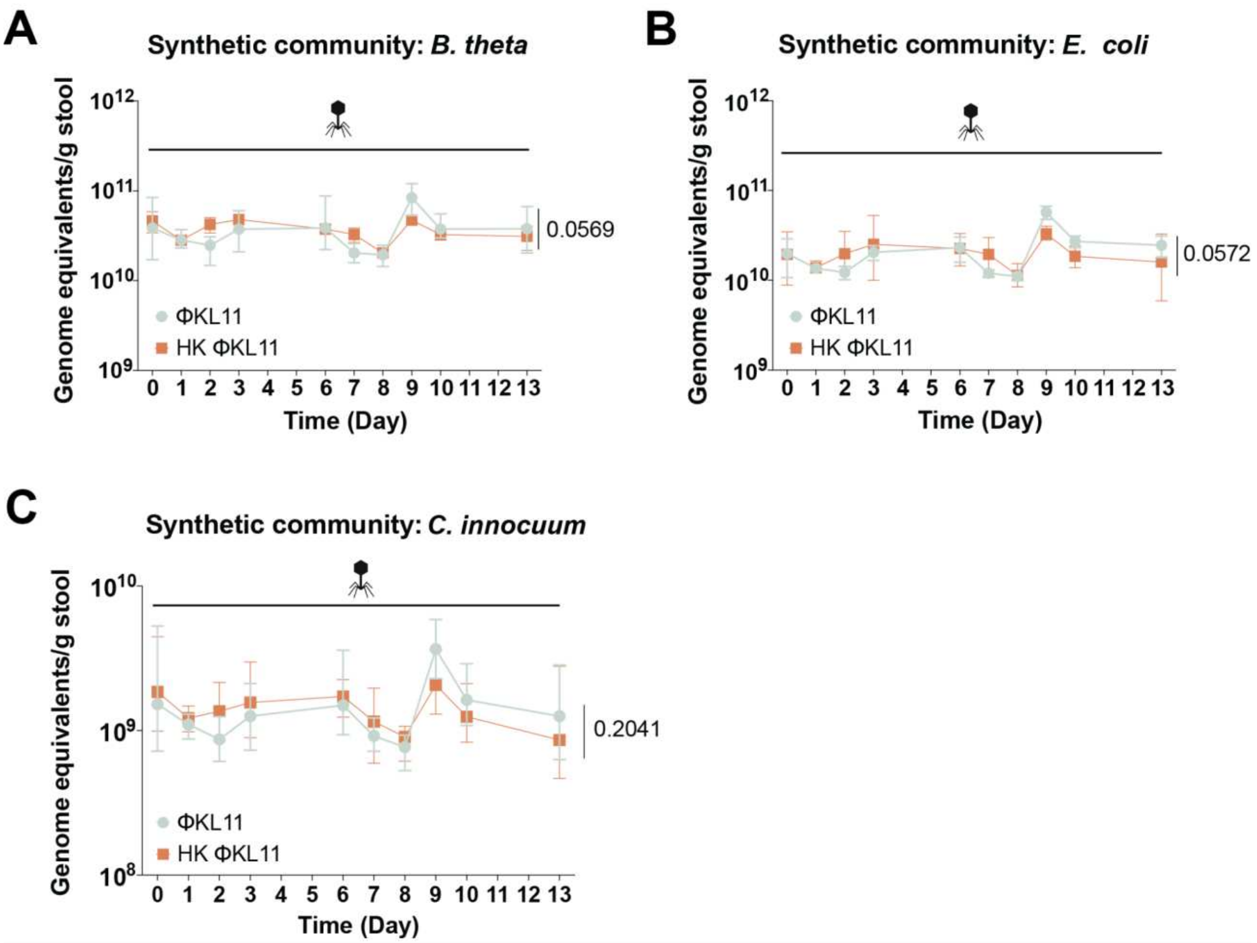

Figure S3. *E. lenta* evades phage predation in a 4-member synthetic microbiota. (A-C) Abundance of **(A)** *B. thetaiotaomicron*, **(B)** *E. coli*, and **(C)** *C. innocuum* measured by qPCR during co-colonization with *E. lenta* DSM2243 (1:1:1:1 initial inoculum; n=5 mice/group). *p*-values, two-way ANOVA.

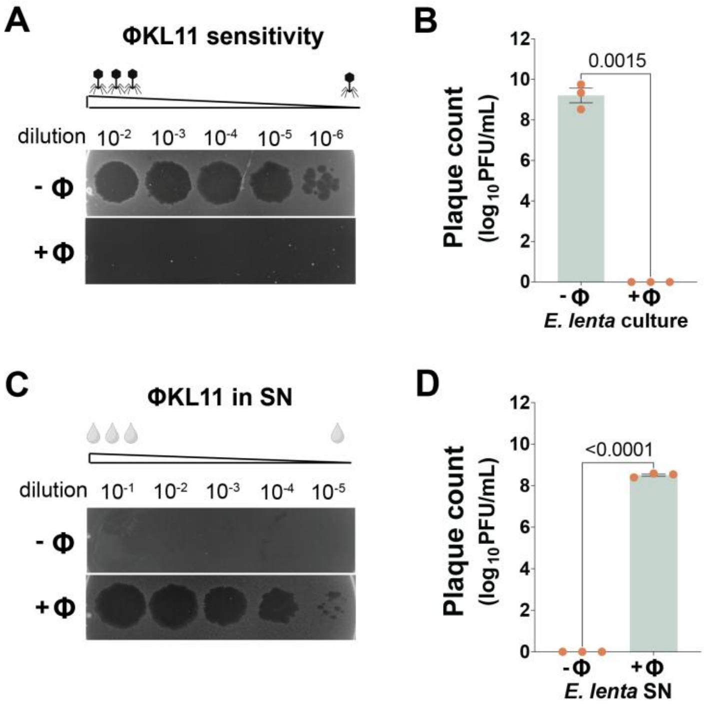

Figure S4. *In vitro* selection for *E. lenta* phage resistance. (A,B) *E. lenta* DSM2243 develops resistance to ΦKL11 after four serial passages in BHI[A] liquid broth in the presence of ΦKL11. **(C,D)** ΦKL11 was detected at high levels (8.51 ± 0.10 $\log_{10}$ units) in the supernatant of ΦKL11-exposed *E. lenta* cultures. Values are mean±SEM. *p*-values, Welch's *t* tests.

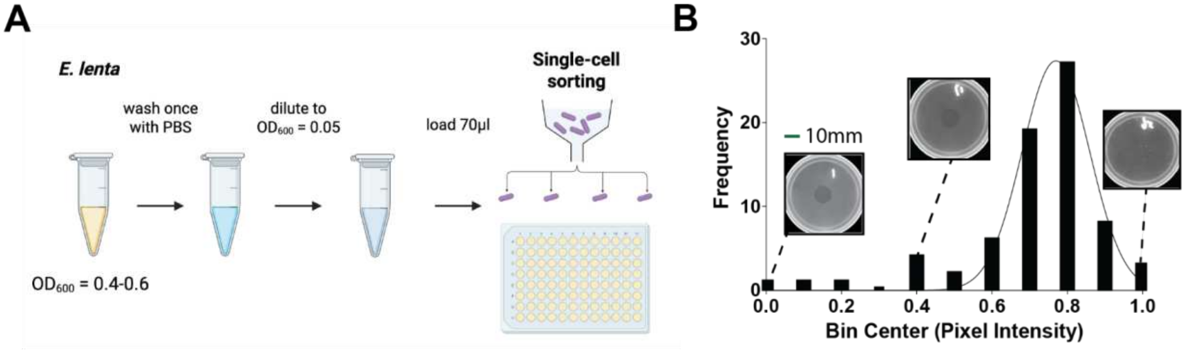

Figure S5. Single cell workflow and distribution of plaque intensities. (A) Workflow diagram. **(B)** Histogram showing the distribution of plaque pixel intensities measured from Φ^{R1}-derived plaques formed on *E. lenta* DSM2243. The x-axis indicates the bin center of normalized pixel intensity, and the y-axis indicates the frequency of plaques within each bin. Representative plaque images corresponding to low, intermediate, and high intensity bins are shown above the histogram. Scale bar, 10mm.

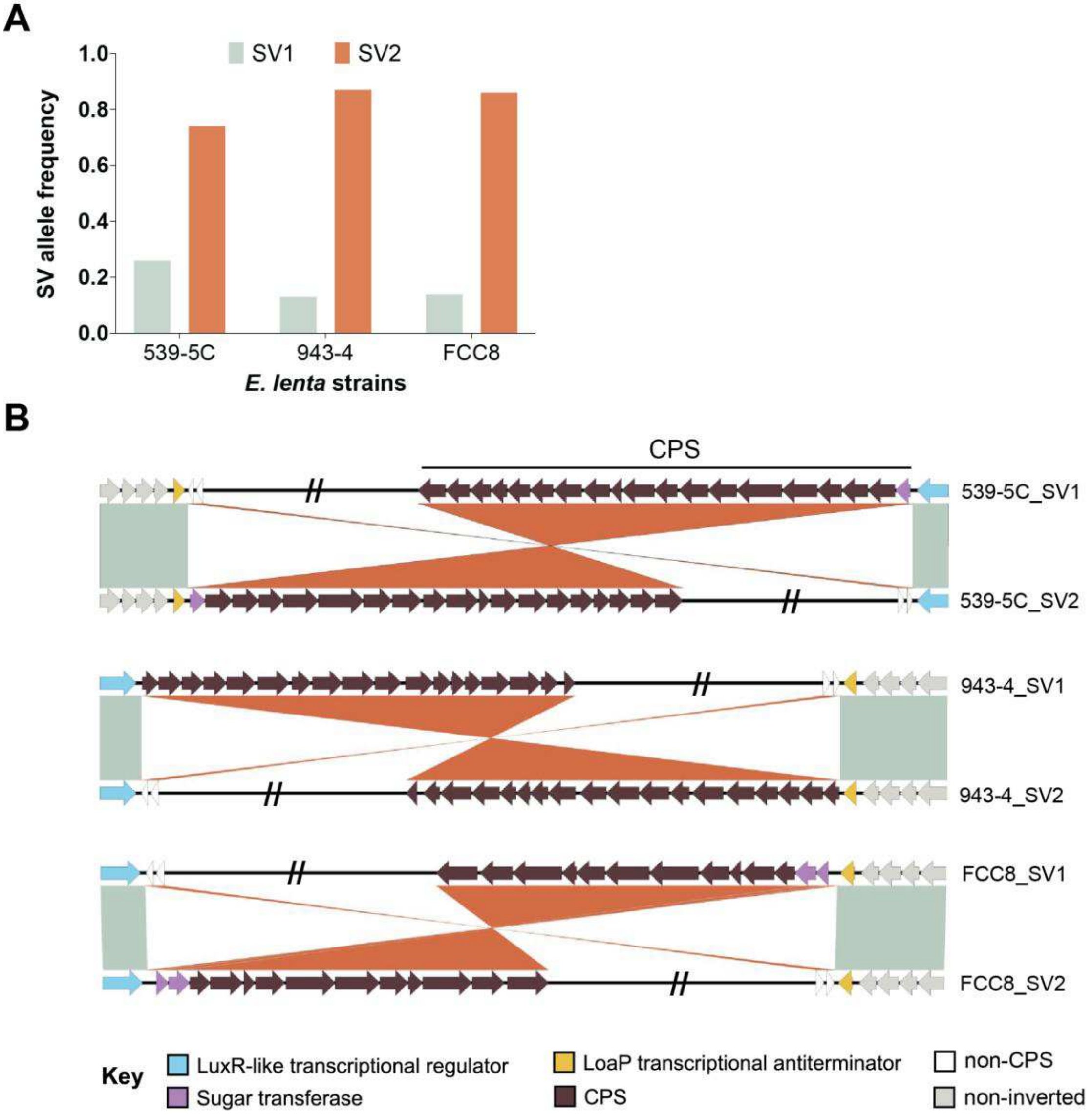

Figure S6. Large genomic inversions are detected in other *E. lenta* strains. (A) Relative abundance of SVs in *E. lenta* strains 539-5C, 943-4, and FCC8, as quantified by long-read sequencing. **(B)** Comparative alignment of the flanking genomic neighborhoods of the inversions identified in *E. lenta* 539-5C SV1/2, 943-4 SV1/2, and FCC8 SV1/2. Orange ribbons denote homologous blocks (crossed ribbons indicate inversions), and mint shading marks syntenic regions in the same orientation. Arrowed boxes represent CDS, highlighting key features of each CPS gene cluster (LuxR-like regulator, sugar transferases, LoaP). Double slashes ("//") denote a 1.9 to 2-Mbp region omitted in SVs.

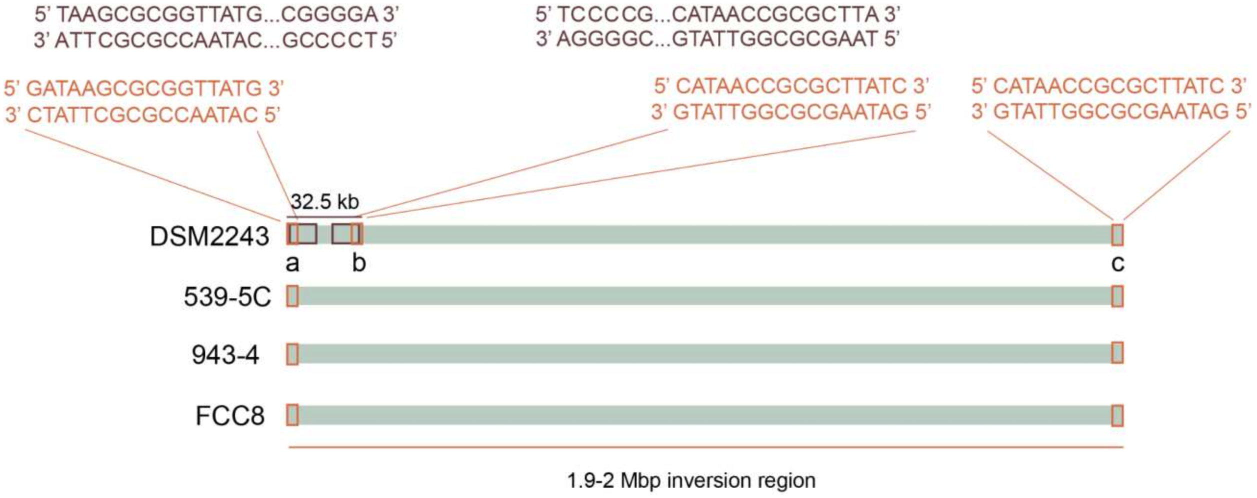

Figure S7. The same 16-bp inverted repeat sequence was identified in *E. lenta* strains harboring large chromosomal inversions. A conserved 16-bp inverted repeat (IR) sequence is observed at inversion junctions a, b, and c in DSM2243 and flanks the ~2-Mbp inversion in strains 539-5C, 943-4, and FCC8. A 39-bp IR sequence is also detected at inversion junctions a and b in DSM2243, flanking the ~32.5 kb inversion. The 16-bp IR overlaps the 39-bp IR and extends 2 bp beyond its boundaries. Orange boxes indicate the 16-bp IR sequences, while brown boxes indicate the 39-bp IR sequences.

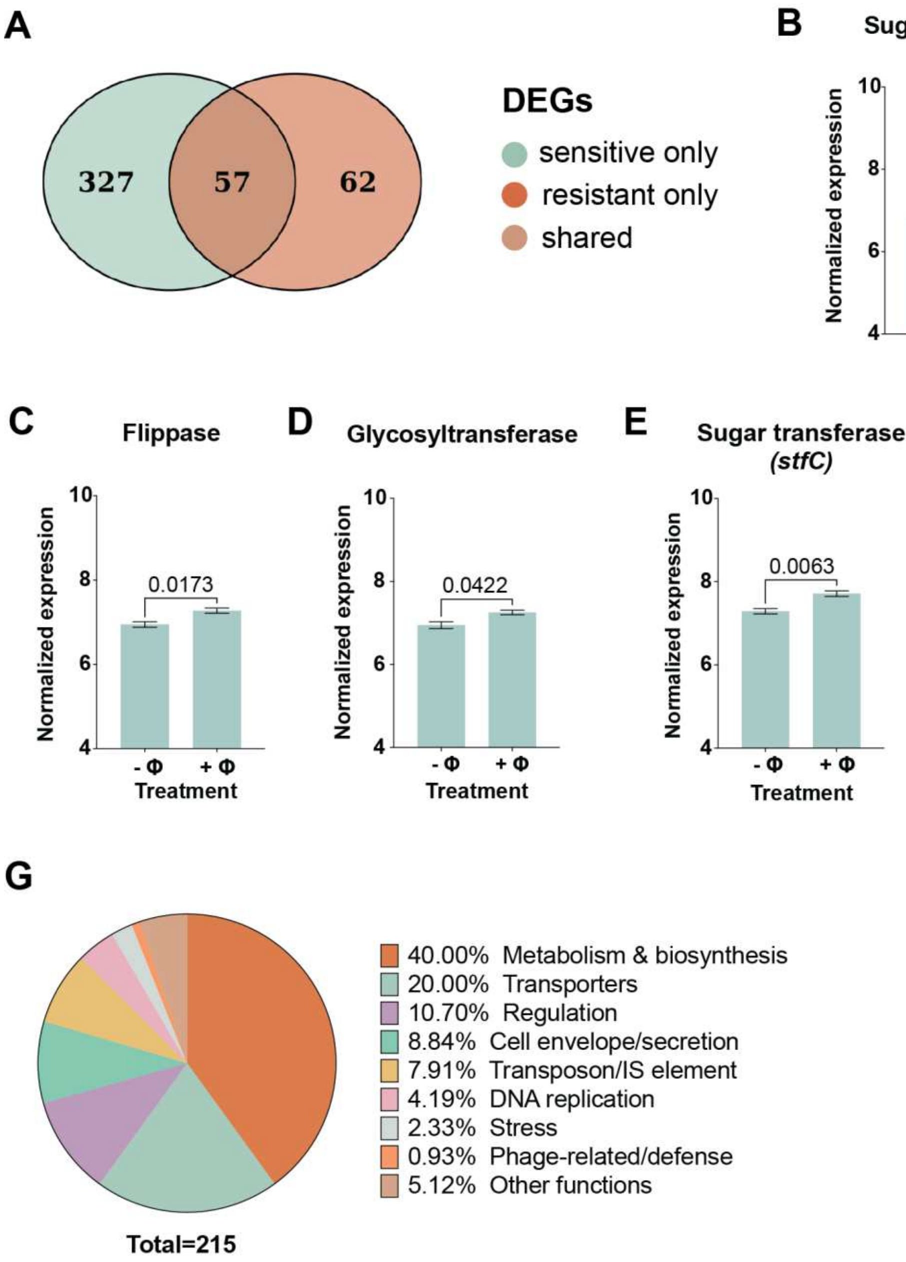
A
327
57
62
DEGs
sensitive only
resistant only
shared
B
Sugar transferase (stfB)
0.0082
Normalized expression
Treatment
- Φ
+ Φ
C
Flippase
0.0173
D
Glycosyltransferase
0.0422
E
Sugar transferase (stfC)
0.0063
F
pseG
0.0065
G
40.00% Metabolism & biosynthesis
20.00% Transporters
10.70% Regulation
8.84% Cell envelope/secretion
7.91% Transposon/IS element
4.19% DNA replication
2.33% Stress
0.93% Phage-related/defense
5.12% Other functions
Total=215

Figure S8. Phage-induced transcriptional responses differ between sensitive and resistant *E. lenta* backgrounds. (A) Venn diagram showing the overlap of differentially expressed genes (DEGs) in response to phage exposure in ΦKL11^S and ΦKL11^R *E. lenta* DSM2243. DEGs were identified by comparing ΦKL11-treated (+Φ) versus no-phage control (-Φ) conditions separately within each host background (p_{adj}<0.05 and |$\log_2$ fold change|≥1). Numbers indicate genes uniquely responsive to phage in sensitive hosts (left), uniquely responsive in resistant hosts (right), or shared between both backgrounds (overlap). **(B-E)** Genes within the CPS2 cluster that are differentially expressed exclusively in phage-sensitive *E. lenta* upon ΦKL11 exposure, shown as DESeq2 variance-stabilized transformed (VST) expression value: **(B)** sugar transferase *stfB* (ELEN_RS12115), **(C)** flippase (ELEN_RS12140), **(D)** glycosyltransferase (ELEN_RS12240), and **(E)** CPS3 sugar transferase *stfC* (ELEN_RS12255). **(F)** *pseG* (ELEN_RS12180) is significantly down-regulated in ΦKL11-treated ΦKL11^S hosts. **(G)** Functional classification of genes up-regulated in ΦKL11^S *E. lenta* following ΦKL11 exposure (total genes=215). Differentially expressed genes in response to phage infection were assigned to functional categories based on predicted annotations, and the pie chart indicates the relative distribution of each category. Genes lacking functional annotations (e.g., hypothetical proteins) were excluded from this analysis. Values are mean±SEM; *p*-values, Welch's *t* tests **(B-F)**.

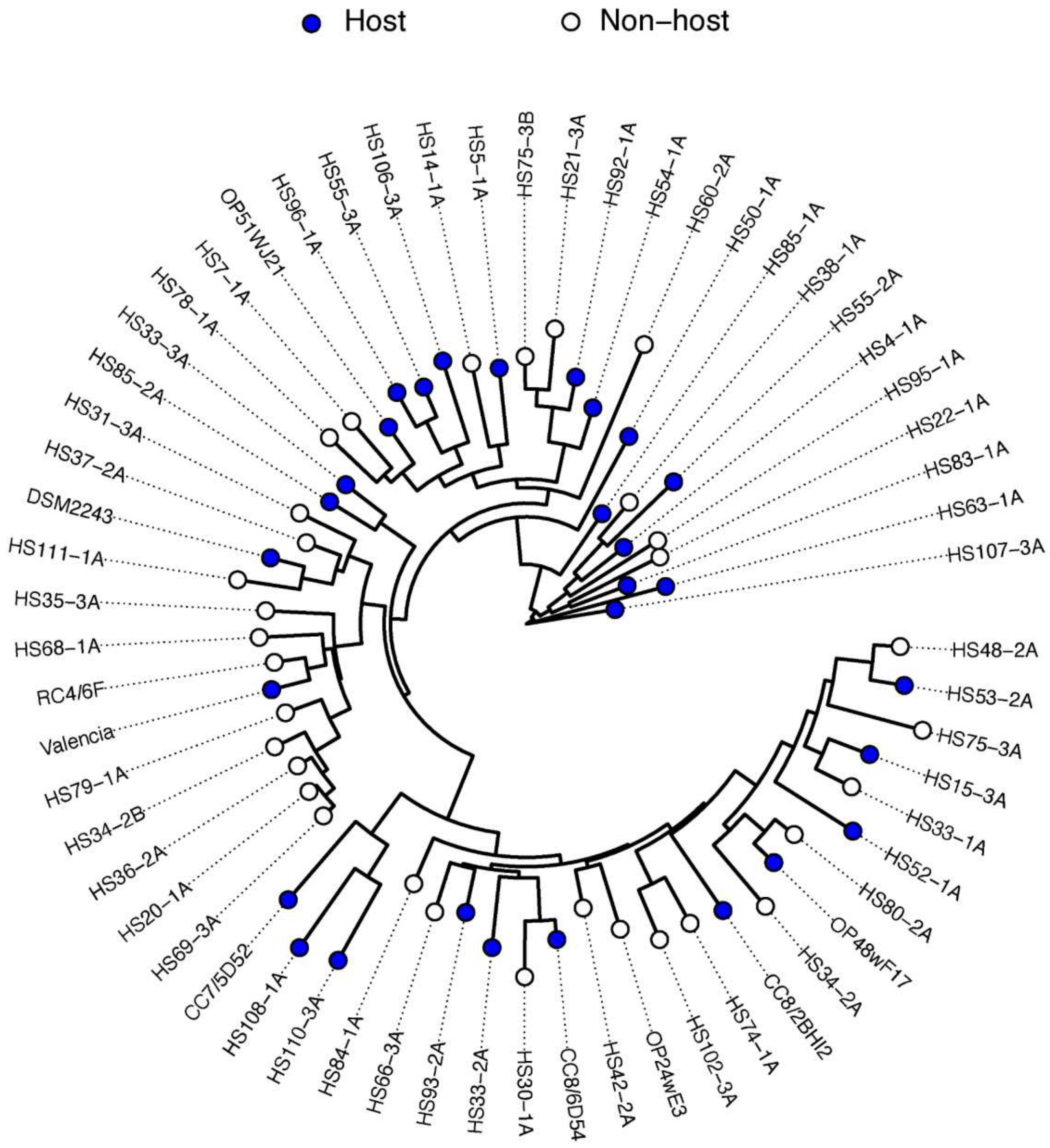

Figure S9. Bacteriophage ΦKL11 has a broad host range. Phylogenetic tree of *E. lenta* strains constructed from core genome alignment. ΦKL11 sensitivity, quantified by plaque assay, is overlaid as a colored dot at each tip, indicating host versus non-host status (n = 61 genomes).

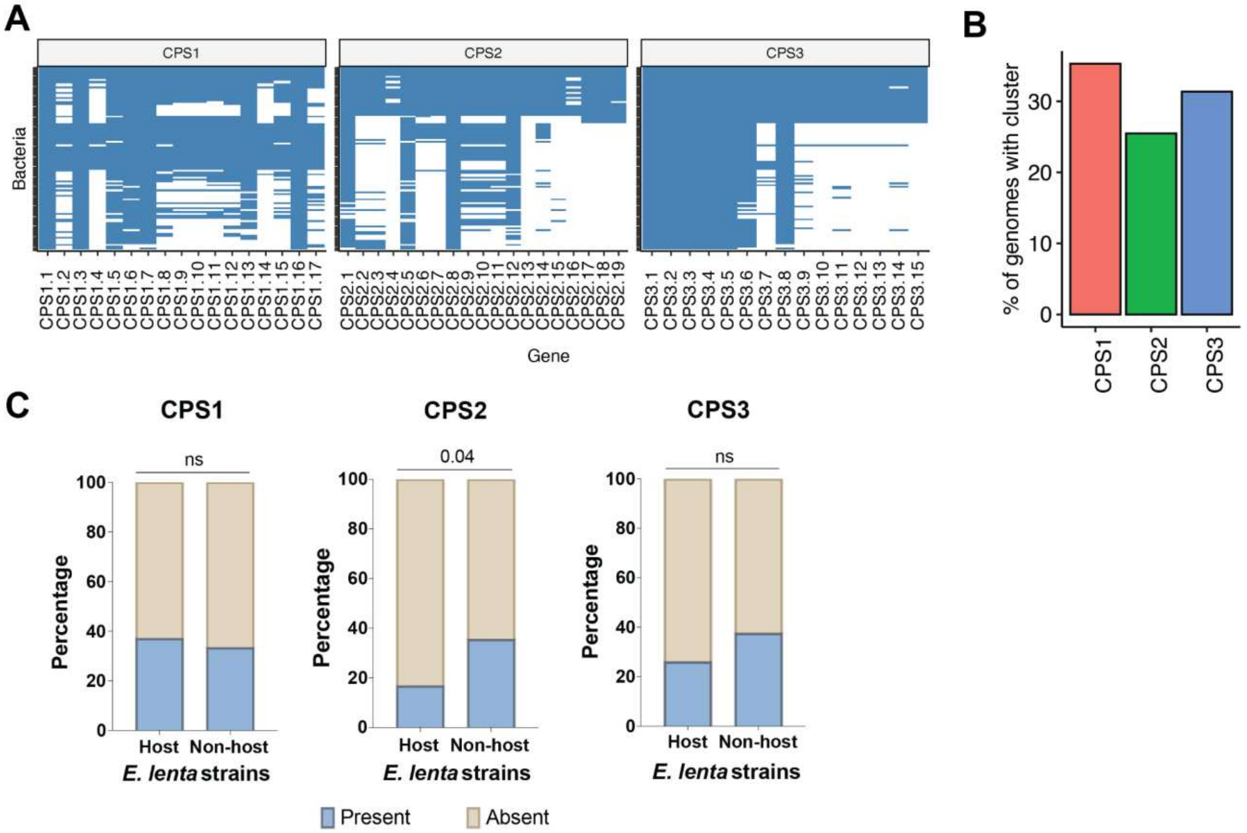

Fig. S10. Prevalence of CPS genes in 102 *E. lenta* genomes. (A) Heatmap showing the proportion of genomes containing genes from each CPS cluster. **(B)** Percentage of *E. lenta* genomes harboring CPS1, CPS2, or CPS3 clusters across the strain collection. A CPS cluster was considered present if ≥85% of the genes belonging to that cluster were detected in the genome, regardless of whether the genes occurred consecutively within a single locus. **(C)** Percentage of ΦKL11 host and non-host strains containing or lacking CPS1, CPS2, or CPS3 clusters using the same ≥85% completeness criterion described in panel B. Bars represent the proportion of strains with a CPS cluster (blue) or lacking the cluster (tan). *p*-values, Fisher's exact test; ns, not significant ($p>0.05$).

SUPPLEMENTAL TABLES

Table S1. Bacteriophages isolated in this study.

Table S2. ΦKL11 genome annotation.

Table S3. Gnotobiotic mouse details.

Table S4. *Eggerthella lenta* sequencing, assembly, and annotation summary.

Table S5. Structural variant (SV) details.

Table S6. Oligonucleotide used in this study.

Table S7. Genes found in each CPS cluster.

Table S8. Differentially expressed genes between phage-resistant and phage-sensitive *E. lenta.*

Table S9. Differentially expressed genes in phage-sensitive *E. lenta* in response to phage.

Table S10. *E. lenta* genome-wide association of orthologous group presence/absence with ΦKL11 susceptibility.

Table S11. *E. lenta* strains included in our comparative genomics analyses.

Supplementary Files

This is a list of supplementary files associated with this preprint. Click to download.

- LentaphageNatMicroSupplTables.xlsx
